# CEDAR OnDemand: a browser extension to generate ontology-based scientific metadata

**DOI:** 10.1186/s12859-018-2247-6

**Published:** 2018-07-16

**Authors:** Syed Ahmad Chan Bukhari, Marcos Martínez-Romero, Martin J. O’ Connor, Attila L. Egyedi, Debra Willrett, John Graybeal, Mark A. Musen, Kei-Hoi Cheung, Steven H. Kleinstein

**Affiliations:** 1 Department of Pathology, Yale School of Medicine, New Haven, CT USA; 2 Interdepartmental Program in Computational Biology and Bioinformatics, Yale University, New Haven, CT USA; 3 Department of Emergency Medicine and Yale Center for Medical Informatics, Yale University School of Medicine, New Haven, CT USA; 4 Stanford Center for Biomedical Informatics Research, Stanford University, Stanford, CA USA

**Keywords:** Ontology, Metadata, CEDAR, FAIR, BioPortal, NCBI, NCBO

## Abstract

**Background:**

Public biomedical data repositories often provide web-based interfaces to collect experimental metadata. However, these interfaces typically reflect the ad hoc metadata specification practices of the associated repositories, leading to a lack of standardization in the collected metadata. This lack of standardization limits the ability of the source datasets to be broadly discovered, reused, and integrated with other datasets. To increase reuse, discoverability, and reproducibility of the described experiments, datasets should be appropriately annotated by using agreed-upon terms, ideally from ontologies or other controlled term sources.

**Results:**

This work presents “CEDAR OnDemand”, a browser extension powered by the NCBO (National Center for Biomedical Ontology) BioPortal that enables users to seamlessly enter ontology-based metadata through existing web forms native to individual repositories. CEDAR OnDemand analyzes the web page contents to identify the text input fields and associate them with relevant ontologies which are recommended automatically based upon input fields’ labels (using the NCBO ontology recommender) and a pre-defined list of ontologies. These field-specific ontologies are used for controlling metadata entry. CEDAR OnDemand works for any web form designed in the HTML format. We demonstrate how CEDAR OnDemand works through the NCBI (National Center for Biotechnology Information) BioSample web-based metadata entry.

**Conclusion:**

CEDAR OnDemand helps lower the barrier of incorporating ontologies into standardized metadata entry for public data repositories. CEDAR OnDemand is available freely on the Google Chrome store https://chrome.google.com/webstore/search/CEDAROnDemand

## Background

Biomedical data are increasingly being deposited in public repositories accompanied by descriptive metadata. These metadata are crucial for facilitating the discovery of the associated datasets and for reproducing the corresponding experiments. Many public data repositories provide web-based forms for researchers to enter metadata describing their datasets as part of the submission process. However, most repositories make limited use of controlled vocabularies in the metadata entry process and, as a result, metadata are often described using inconsistent terminologies [1]. This lack of standardization makes it difficult to access, find, interoperate, and reuse the datasets, and—crucially—to understand how the associated experiments were performed. Improvements are needed to make these datasets more FAIR (Findable, Accessible, Interoperable, and Reusable) [2]. The use of terms from controlled terminologies and ontologies can provide an important first step for creating FAIR metadata descriptions [3].

A wide array of ontology-based services have been developed in order to promote scientific data interoperability and reusability in biomedicine through the use of standard terminologies. These include BioPortal [4], the Ontology Lookup Service (OLS) [5], EBI Zooma [6], and NCBO Annotator and Recommender [7, 8]. In addition, data (metadata) standardization efforts have been established by different communities to ensure sufficient amount of information (metadata) be provided for reporting results in a way that facilitates reproducibility such as MIAIME (Minimum Information for Reporting Microarray Experiment) [9], MiAIRR (Minimal Information about Adaptive Immune Receptor Repertoire) [10, 11] and MIBBI (Minimum Information for Biological and Biomedical Investigations) [12]. The Center for Expanded Data Annotation and Retrieval (CEDAR) [13] has leveraged existing data standards and the ontologies available at BioPortal to develop the CEDAR Workbench with the goal of creating semantically rich metadata. A user either can create a new template (web form) or can use existing ontology-controlled templates to author standardized metadata within CEDAR Workbench. An example employing CEDAR Workbench for customized data submission is [14]. Expanding CEDAR’s approach of metadata creation outside of its environment, we have incorporated BioPortal ontologies and web services to develop a *decentralized metadata authoring tool* called “CEDAR OnDemand”. CEDAR OnDemand is a platform-independent program running as a web browser extension designed to help creating standardized metadata in repository-native web forms. The key advantage of this approach is that it enables users to seamlessly enter ontology-based metadata into existing web forms without requiring the individual repositories to provide these services.

## Implementation

The CEDAR OnDemand script has been developed as a Google chrome browser extension [15] (a browser extension is essentially a small software program that can access contents of a web page, modify it and can enhance the functionality of a web browser). It is powered by the NCBO Annotator [7] and Recommender [8] Web services and facilitates users to suggest entry-time ontology controlled metadata to fill up web forms. After installation, the extension will appear as an icon on the chrome extension bar (upper right side of the browser). It is designed to be manually toggled on upon entry of a web form (it can be toggled off later if needed). Although CEDAR OnDemand can be programmed to be auto-activated, we used the manual activation method to minimize the system memory usage and to protect users from browser-based security attacks [16]. The extension operates in three phases (described below) that are initiated when a user visits a new web-based (metadata) entry form.

### Identification of data entry fields

**Fig. 1:**
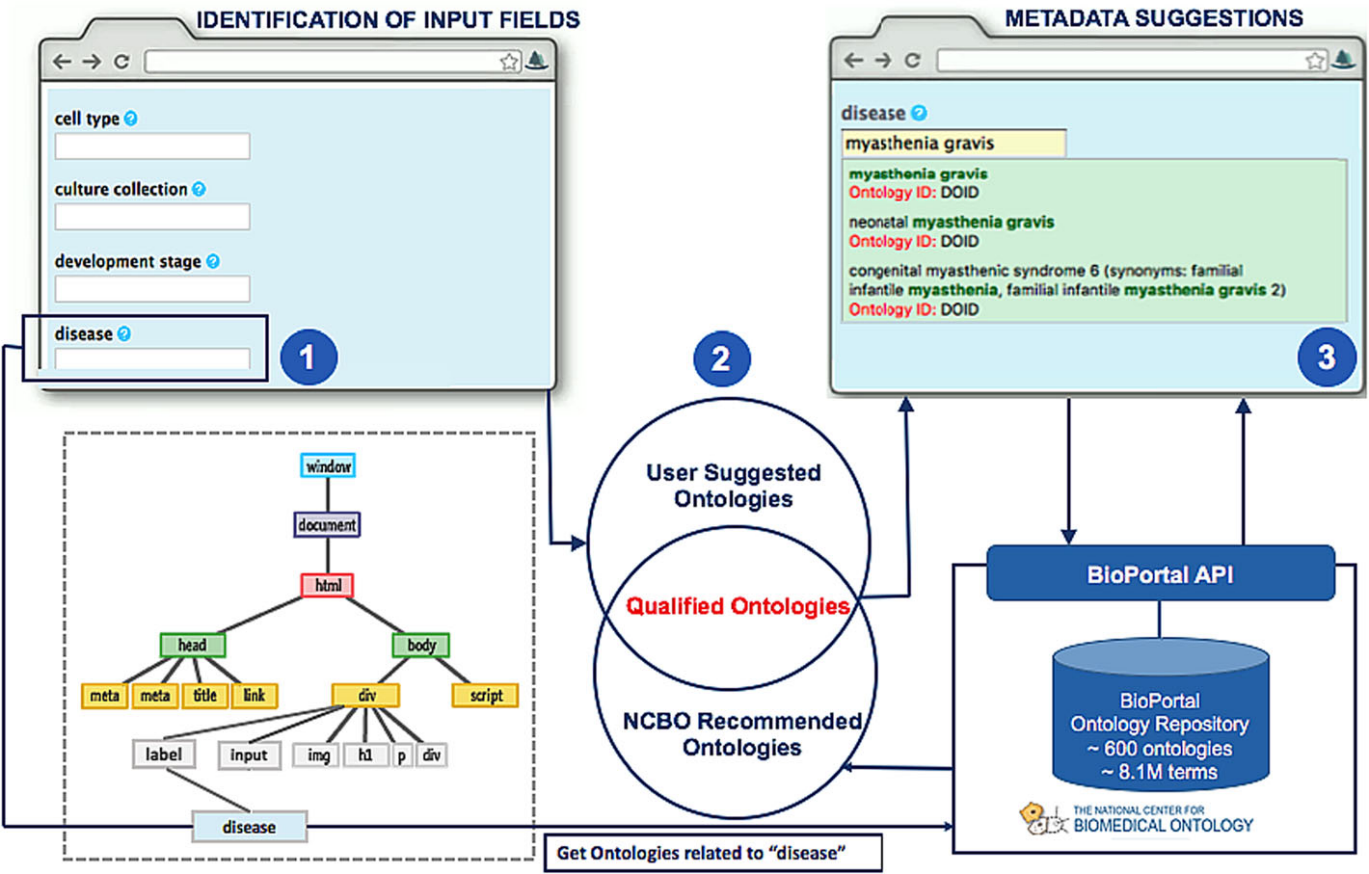
CEDAR OnDemand Workflow. (1) CEDAR OnDemand is installed as a Google Chrome browser extension. When activated by the user (by toggling the icon), the web page in the browser will be analyzed. Users can customize a list of suggested ontologies used by CEDAR OnDemand and any point through a dialogue box with dropdown list (2) An HTML tags detection script identifies the text fields by analyzing the HTML INPUT tags and fetches their labels. Fetched labels are then passed to the NCBO to get the related ontology recommendations. This BioPortal ontology recommendation list is compared to the user suggested ontology list to find the qualified ontologies (3) CEDAR OnDemand associates the qualified ontology list to the detected input fields. Subsequently, NCBO Annotator service is invoked to match field values with the qualified set of ontologies to suggest ontology-based metadata

To detect data entry fields, the web page is analyzed to identify text input fields and the associated field labels (Fig. 1, left side). CEDAR OnDemand parses the content of a web page into the document object model (DOM) [17], which defines the content, structure and style of an HTML document (Fig. 1, left panel treeview). The current implementation of CEDAR OnDemand recognizes the standard INPUT fields (HTML5 and previous versions) and their associated labels (HTML5 element). The recognized fields are highlighted with light yellow color. The metadata entry of the detected input fields will be controlled by the list of ontologies chosen by the qualified ontologies.

### Ontologies recommendation algorithm

The CEDAR OnDemand ontology recommendation algorithm is designed to recommend ontologies relevant to each input field listed in a webform from the BioPortal [4] ontologies. CEDAR OnDemand takes each field label as input (as shown in Label 2 in Fig. 1) to the NCBO Recommender 2.0 service [8] to get a list of BioPortal ontologies (containing terms matching the field label). Moreover, a user can also define ontologies through a dialogue box which appears by toggling the CEDAR OnDemand extension. The CEDAR OnDemand algorithm takes the intersection of the set of user-defined ontologies and that of ontologies recommended automatically (by the NCBO recommender) to produce the set of qualified ontologies for each field. These field-specific qualified ontologies are then linked to each input field in a web form. If the intersection is an empty set, then the full user-defined list is used for as the qualified ontologies for controlling the field entry. By default, the user-defined list includes six ontologies: ChEBI Ontology [18], Human Disease Ontology (DOID) [19], Gene Ontology (GO) [20], Ontology for Biomedical Investigations (OBI) [21], Phenotypic Quality Ontology [22], Protein Ontology (PR) [23] (Fig. 1, Label 2). Not only do these ontologies cover a broad range of biological domains, but they are also ranked among the top ten by OBO Foundry in terms of their compliance to ontology best practice [24]. The user may change the default ontology list by adding/removing ontologies anytime during the metadata entry process. In its default behavior CEDAR OnDemand works fully automatically and does not require an ontology input from the user. However, customizing the default ontology list may help the user to get domain-specific metadata suggestions.

### Ontology association and auto-completion of metadata

To associate ontology terms (e.g., “myasthenia gravis” from DOID) with the field entry (e.g., disease), CEDAR OnDemand matches the term(s) entered by the user with the terms defined in the qualified ontologies (Fig. 1, Label 3). This is done by invoking the NCBO Annotator web service [8] through AJAX (asynchronous JavaScript and XML) call [25]. AJAX communicates with NCBO BioPortal server [26] asynchronously (in the background) through XMLHttpRequest Object to send and retrieve the data. This asynchronous communication model of CEDAR OnDemand enables entry-time suggestion for ontology controlled metadata entry. The NCBO Annotator returns a ranked list of ontology term matches for the user to choose.

## Results

We tested CEDAR OnDemand by entering metadata using the NCBI human BioSample web form^1^ [27]. In this use case, we first extended the user defined ontology list by adding several field-specific ontologies identified through NCBO recommender: Cell ontology (CL) [28], Cell Line Ontology (CLO) [29], NCI thesaurus NCIT [30], NCBI Taxonomy ontology NCBITAXON [31], and Uber Anatomy Ontology (UBERON) [32]. The NCBI human BioSample web form contains twenty-one text input fields. CEDAR OnDemand suggested eight ontologies based on the input fields in the NCBI human BioSample web form. After intersection with the user defined ontologies (extended list), the final ontology list recommended by the CEDAR OnDemand includes: NCI thesaurus NCIT [30], Cell Ontology [28], Cell Line Ontology [33], (UBERON) [32], Human disease Ontology [19], Gene Ontology (GO) [20] and OBI [21] (See Table 1). Controlled vocabularies do not make sense for some text fields, such as *“Sample Name”, “Age” and “isolate”.* Therefore, CEDAR OnDemand allows the user to override ontology suggestions for all fields with the user-defined entries. CEDAR OnDemand provides the field's specific metadata suggestion controlled by ontologies. Thus, users are no longer entering free text but they are instead using standardized ontology terms. An auto-completion feature is provided at runtime through a drop-down list. As an example (Fig. 1, Label 3), CEDAR OnDemand suggests *“myasthenia gravis”* as controlled term (defined in DOID) for the disease field.

**Table 1:** CEDAR OnDemand Qualified Ontologies for each NCBI BioSample Field

Field names	Qualified ontologies
Sample Name	Ontology for Biomedical Investigations (OBI), National Cancer Institute Thesaurus (NCIT)
Organism	National Cancer Institute Thesaurus (NCIT)
Isolate	National Cancer Institute Thesaurus (NCIT)
Age	National Cancer Institute Thesaurus (NCIT)
Biomaterial Provider	National Cancer Institute Thesaurus (NCIT)
Tissue	Uber Anatomy Ontology (UBERON), Ontology for Biomedical Investigations (OBI), National Cancer Institute Thesaurus (NCIT), Cell Ontology (CL), Cell Line Ontology (CLO)
Cell line	Cell Line Ontology (CLO), Ontology for Biomedical Investigations (OBI), National Cancer Institute Thesaurus (NCIT)
Cell subtype	Cell Ontology (CL), Gene Ontology (GO), National Cancer Institute Thesaurus (NCIT)
Cell type	Cell Ontology (CL), Cell Line Ontology (CLO), National Cancer Institute Thesaurus (NCIT)
Culture Collection	National Cancer Institute Thesaurus (NCIT)
Development Stage	Gene Ontology (GO), National Cancer Institute Thesaurus (NCIT)
Disease	Human Disease Ontology (DOID), Cell Line Ontology (CLO), Ontology for Biomedical Investigations (OBI), National Cancer Institute Thesaurus (NCIT)
Disease Stage	Human Disease Ontology (DOID), Cell Line Ontology (CLO), Ontology for Biomedical Investigations (OBI), National Cancer Institute Thesaurus (NCIT)
Ethnicity	National Cancer Institute Thesaurus (NCIT)
Health state	National Cancer Institute Thesaurus (NCIT)
Karyotype	National Cancer Institute Thesaurus (NCIT)
Phenotype	Ontology for Biomedical Investigations (OBI), National Cancer Institute Thesaurus (NCIT)
Population	Ontology for Biomedical Investigations (OBI)
Race	National Cancer Institute Thesaurus (NCIT)
Sample type	National Cancer Institute Thesaurus (NCIT)
Treatment	Ontology for Biomedical Investigations (OBI), National Cancer Institute Thesaurus (NCIT)

Field Names column lists the Human Sample attributes of NCBI BioSample. Qualified Ontologies are the ontologies which CEDAR OnDemand algorithm recommends

## Discussion

Although many public repositories, such as those run by the NCBI, provide easy-to-use tools and interfaces for entering and querying metadata, scientists who upload their datasets are generally not constrained to use standard terminologies when they define the necessary metadata. As a result, metadata are often described using inconsistent terminologies, limiting scientists’ ability to access, find, interoperate and reuse the datasets and to understand how the experiments were performed. Scientific data analysis or mining [34] often requires multiple datasets to be integrated within a single repository or across multiple repositories. Such integration would be easier if the datasets and their metadata were identified globally, described using standardized terminologies, and available in a standardized machine readable format. A common semantic schema [35] among different studies and data sources can be achieved by associating relevant ontology classes with each study's metadata. Despite the free availability of ontology resources [26, 36], only few repositories (e.g., IEDB -The immune epitope database [37]) and frameworks (e.g., SEBI-Semantic enrichment of biomedical Images [38, 39]) have integrated ontologies or structured controlled lists within their framework to collect standardized metadata.

PubMed uses Medical Subject Headings [40] as a controlled vocabulary for indexing and searching biomedical literature. Meshable [41] highlights an important issue in PubMed literature searching. In PubMed, biologists can use MeSH terms as queries to get the precise results. However, these are rarely used, and there is no convenient way to author standardized MeSH terms as queries. Through CEDAR OnDemand, users can suggest MeSH Ontology [42] replacing the default user-defined list and can get entry-time query suggestions from the MeSH controlled vocabulary.

CEDAR OnDemand has the potential to improve the FAIRness and overall quality of metadata to the available repositories. However, the current infrastructure has some limitations. For instance, the diversity in the input field coding schemes (e.g., *<div, <inputfield and < text*) limits the HTML tags detection script when there are custom-build tags are used to define the input fields. Our script identifies the standard HTML5 tags, *Label* was introduced in HTML5. However, input tag was present at the very beginning (i.e., *<input type = “text”*) to represent an input field. Though CEDAR OnDemand works with web forms designed in HTML4 or with older versions, the ontology recommendation algorithm does not make use of the field associated (labels) information for ontology recommendation in these cases, relying instead on the users suggested ontology list.

A key component of CEDAR OnDemand is the ability to analyze context and suggest appropriate ontologies for each particular field. The current qualified ontology selection process relies on NCBO ontology recommender service [8] and the user’s suggested ontology list. We have proposed this scheme as the NCBO recommended ontology list can be very long, and may not always recommend ontologies that are specific to a user’s particular domain. Allowing users to customize a set of suggested ontologies helps to address both these issues. Ideally, using the field context along with NCBO recommender would be able to identify and rank all of the relevant ontologies. In practice, it can be difficult to get sufficient context just from the web page and text surrounding a field. Even if enough context is present, it may be technically difficult to extract. For example, the web interfaces for some repositories have been designed using older versions of HTML and some with custom HTML tags.

We have tested CEDAR OnDemand with the latest Chrome version (59.0.3054) on Mac and Windows. The core of CEDAR OnDemand is a based on Javascript and should work with any version of chrome browser with its default setup on Windows, Mac OS and Linux operating systems. We are exploring the possibility of supporting other types of browsers (e.g., Firefox and Microsoft Edge).

## Conclusions

CEDAR OnDemand is a chrome browser extension that enables users to seamlessly enter ontology-controlled metadata using existing web-based submission forms provided by metadata repositories. The use of controlled vocabularies for entering metadata can help improve the quality of metadata submitted to repositories and ultimately contributes to the creation of FAIR data.

## Availability and requirements

Availability: https://chrome.google.com/webstore/search/CEDAROnDemand

Code Availability: https://github.com/ahmadchan/CEDAROnDemand

Project name: CEDAR OnDemand.

Operating system(s): Operating system independent works within web browser.

Programming language: Javascript.

License: GPL.

Any restrictions to use by non-academics: none.

## Footnotes

1
https://submit.ncbi.nlm.nih.gov/subs/biosample/
